# Heterogeneity of glioblastoma stem cells in the context of the immune microenvironment and geospatial organization

**DOI:** 10.3389/fonc.2022.1022716

**Published:** 2022-10-19

**Authors:** Aryeh Silver, Diana Feier, Tanya Ghosh, Maryam Rahman, Jianping Huang, Matthew R. Sarkisian, Loic P. Deleyrolle

**Affiliations:** ^1^ Department of Neurosurgery, Adam Michael Rosen Neuro-Oncology Laboratories, University of Florida, Gainesville, FL, United States; ^2^ Preston A. Wells, Jr. Center for Brain Tumor Therapy, University of Florida, Gainesville, FL, United States; ^3^ Department of Neuroscience, McKnight Brain Institute, University of Florida, Gainesville, FL, United States

**Keywords:** Glioblastoma, cancer stem cells, tumor microenvironment, immune landscape, heterogeneity, cell interactions, spatial profiling

## Abstract

Glioblastoma (GBM) is an extremely aggressive and incurable primary brain tumor with a 10-year survival of just 0.71%. Cancer stem cells (CSCs) are thought to seed GBM’s inevitable recurrence by evading standard of care treatment, which combines surgical resection, radiotherapy, and chemotherapy, contributing to this grim prognosis. Effective targeting of CSCs could result in insights into GBM treatment resistance and development of novel treatment paradigms. There is a major ongoing effort to characterize CSCs, understand their interactions with the tumor microenvironment, and identify ways to eliminate them. This review discusses the diversity of CSC lineages present in GBM and how this glioma stem cell (GSC) mosaicism drives global intratumoral heterogeneity constituted by complex and spatially distinct local microenvironments. We review how a tumor’s diverse CSC populations orchestrate and interact with the environment, especially the immune landscape. We also discuss how to map this intricate GBM ecosystem through the lens of metabolism and immunology to find vulnerabilities and new ways to disrupt the equilibrium of the system to achieve improved disease outcome.

## Cancer stem cells (CSCs) in GBM

**Figure 1:**
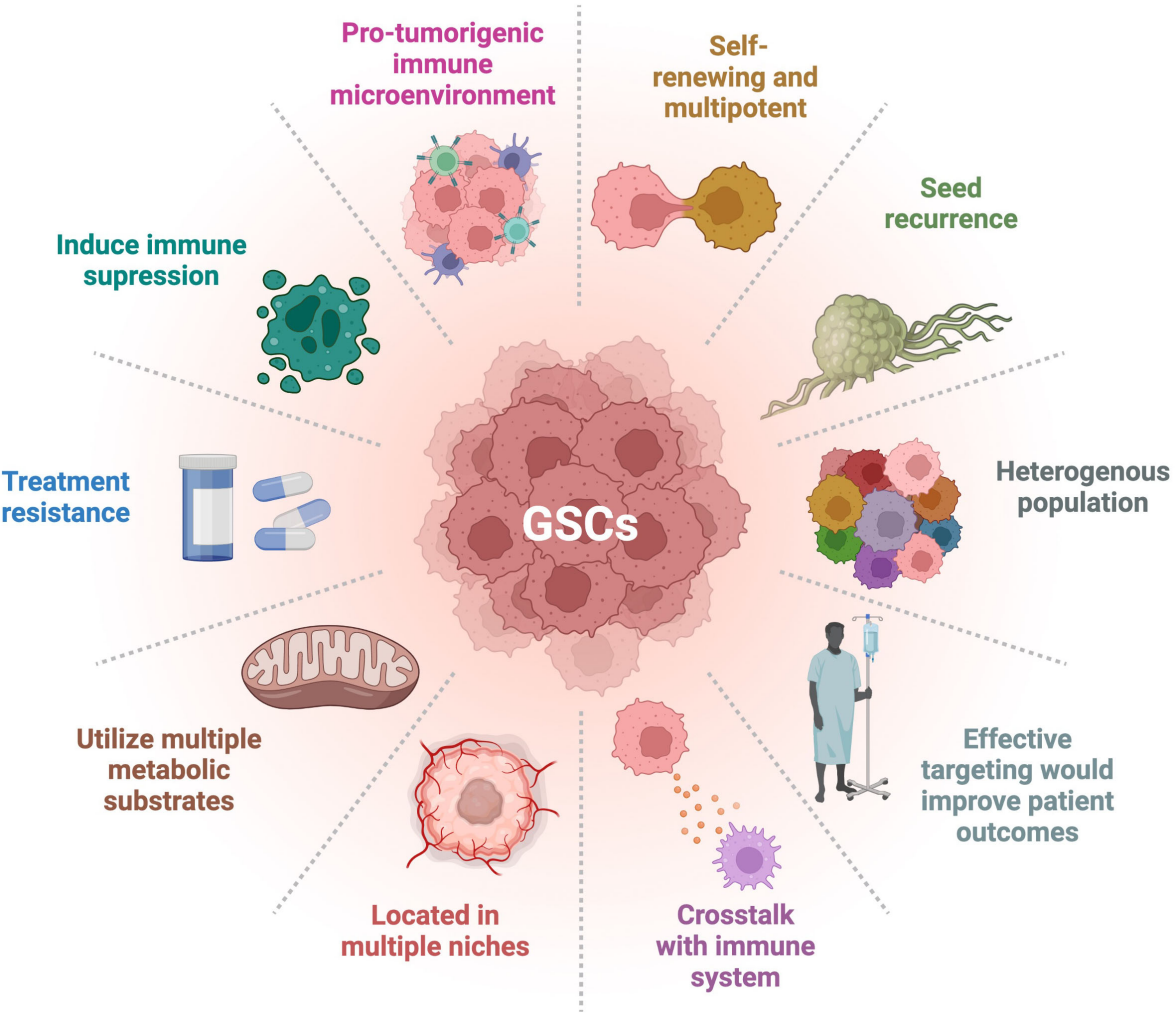
Hallmarks of GSCs. GSCs have many distinct characteristics that differentiate them from other types of tumor cells. Their degree of stemness is related to the hallmarks presented. Adapted from “Hallmarks of Cancer: Circle”, by BioRender.com (2022). Retrieved from https://app.biorender.com/biorender-templates.

Glioblastoma (GBM) is a very aggressive and incurable disease with a median survival of about 15 months and a 5-year survival rate of only 5.5% (1–3). This dismal prognosis may be due to lack of adequate treatment and targeting of glioma stem cells (GSCs). Cancer stem cells (CSCs) have been observed and described in a number of cancers, including GBM, and their hallmarks are presented in **Figure 1**. GSCs are self-renewing, tumorigenic cells that drive tumor formation, progression, and ultimately disease recurrence (4, 5). GSCs are multipotent and can differentiate along multiple lineages into progenies with varying characteristics that create different niches, heavily contributing to the heterogeneity found in GBM (6). This heterogeneity is associated with poor prognosis and treatment resistance (5, 7, 8). GSC is a collective term for a wide array of heterogeneous cells that share certain phenotypic characteristics and functional properties. There is no clear dichotomy between non-GSCs and GSCs; rather, there appears to be a cellular spectrum that spans multiple cell types or states (9). There is no consensus over a strict set of characteristics delineating GSCs. These cells overexpress several markers including CD133, CD44, CD15, A2B5, PTPRZ1, ITGB8, L1CAM, SOX2, and Nestin, which are common but not defining (5, 10–13). GSCs can also be classified based on their behavioral and functional properties, such as enhanced sphere forming ability and being slow-cycling (14). Glycerol-3-phosphate dehydrogenase 1 (GPD1), a key player in oxidative phosphorylation (OxPhos), has been purported to be enriched in dormant GSCs (12). Slow-cycling GSCs were also described to exhibit enhanced mitochondrial activity and lipid metabolism (14). However, not all GSCs express these markers or exhibit these features, which are not exclusive to these cells. For instance, it was shown that CD133 negative GBM cells harbored stem cell properties, such as tumor forming ability (9). Currently, much effort is being made by the cancer stem cell community to standardize and innovate assays for better characterization and targeting of CSCs. GSCs are important factors in driving the progression of GBM through their roles in immune suppression, preventing immune cells from sufficient uptake of nutrients such as glucose and oxygen, promoting angiogenesis, and increased invasion and metastatic abilities (4). In creating a more immunosuppressive environment, GSCs have been seen to inhibit T cell proliferation and cytotoxic T cell activation, as well as secrete factors such as IL-10 and TGFβ, which suppress the tumor-killing function of macrophages (15). GSCs exhibit specific metabolic regulations allowing them to outcompete neighboring cells for nutrients, such as glucose through upregulation of glucose transporters (16), or lipids *via* the overexpression of fatty acid transporters and binding proteins (14). In addition, the hypoxic conditions of the TME promote GSC survival by furthering the stem-like state through glycosylation of CD133 which plays a role in anti-hypoxia-mediated apoptosis (17). GSCs can express higher levels of proangiogenic growth factors such as vascular endothelial growth factor (VEGF) and can transdifferentiate into pericytes, contributing to vascular structure (18). GSCs also display greater infiltration capacities and increased migratory abilities through engaging in the epithelial-mesenchymal transition **(**EMT) pathway (14, 19). By utilizing these specific characteristics, these cells can shape a supportive TME, thereby promoting cancer progression and leading to worse disease outcome (19).

## Heterogeneity of GSCs in GBM

One of the hallmarks of GBM is its cellular heterogeneity (**Figure 1**). This is reflected in the heterogeneity of the GSC makeup of a tumor as well (5, 20, 21). Multiple populations of GSCs have been defined based on the expression of various markers (5). For instance, CD133 is commonly used as a marker for GSCs. However, there has been evidence for CD133 negative stem-like cells (22). Both positive and negative cells can give rise to cells that express markers of neurons, astrocytes, and oligodendrocytes, demonstrating multipotency with the ability to give rise to different cell lineages (**Figure 1**). Furthermore, both subtypes were identified as similarly tumorigenic, although the proliferation index for the CD133 negative line was lower (22). Different cell types can express one or a combination of CSC markers, suggesting a diverse range of stemness within GSC populations (5, 23). Expression of other markers, such as EGFRvIII, can increase stemness of a cell population as well (20, 24). Tumor samples have been found to contain multiple CSC types, each exhibiting distinct characteristics, such as differing abilities to give rise to multinucleated giant cells (25, 26). These separate subclones of GSCs also showed different responses and resistance to treatments. Another study demonstrated that different clones have varying sphere-forming abilities, relating to their proliferation and self-renewal (27). These different GSCs can therefore contribute specifically and differently to the tumor presentation and progression.

The diversity of GSC populations can be explained by their spatial and cellular origin, which can include stem cells, progenitor cells, and differentiated cells (6, 28). Interestingly, heterogeneity has also been noted in adult neural stem cells (aNSCs), with a spectrum depending on the developmental stages and spatial location of these cells, impacting their self-renewing capabilities and multipotency (29, 30). Drawing from the idea that GSCs may arise from aNSCs, this concept can be extrapolated to explain that they share these heterogeneous features as well. GSCs vary in phenotypes and properties, and their stem-like states has been described to change over time (23). GSCs exist along a fluid spectrum of stemness with a dynamic cellular composition and geospatial distribution regulated by intrinsic and extrinsic mechanisms and dependent on the local microenvironment, the stage of the disease, and the exposure to treatments (20, 31, 32). Heterogeneity in GBM is also reflected by the existence of different transcriptional and molecular subtypes identified by bulk RNA sequencing. GBM has been classified into four subtypes (proneural, neural, mesenchymal, and classical) characterized by specific genetic aberrations and gene expression of EGFR, NF1, and PDGFRA/IDH1 (33). Not only can these subclasses vary between patients, they can also co-exist in different areas within the same tumor. Subsequent single cell RNA sequencing studies showed that distinct cells of a given tumor can engage programs recapitulating each distinct subtype (34–36). The subtypes can also change between primary and recurrent tumors with about 65% of primary GBM switching subclass after recurrence (37). Furthermore, the cell of origin and initiating anatomical location may influence the GBM subtype (38–42). For instance, the mesenchymal and proneural subtypes are suggested to originate from an astrocytic lineage and oligodendrocyte precursor cell lineage, respectively (39, 41). Interestingly, each subtype is associated with a different treatment sensitivity and immune signature (36, 43–46). Additionally, distinct GSC signatures are identified in the different GBM subclasses. The mesenchymal subtype is, for example, characterized by low expression of CD133 and high levels of expression of CD44, YKL40, BMI1, ALDH1A3, TWIST1, SNAI1-2, TGFB1, STAT3, and CD248, whereas the proneural subtype is defined by GSCs with high expression of CD133, OLIG2, SOX2, and EZH2 (47–49). Mesenchymal GSCs tend to localize to hypoxic and necrotic areas, while proneural GSCs are mostly found in the perivascular regions (48). More recently, using single cell RNA sequencing, Neftel and colleagues demonstrated the presence of four different malignant cellular states including neural-progenitor-like (NPC-like), oligodendrocyte-progenitor-like (OPC-like), astrocyte-like (AC-like), and mesenchymal-like (MES-like) (50). The frequency of each state is correlated to genetic alterations in CDK4, PDGFRA, EGFR, and NF1, with each alteration favoring a particular state. Tumors enriched for the AC-like and MES-like states correspond to the bulk defined subtypes, classical and mesenchymal, respectively, whereas the proneural subtype corresponds to the combination of the OPC-like and NPC-like states. Finally, the neural subclass reflects a dominance of nonmalignant oligodendrocytes and neurons. The immune environment differs between subtypes as well. For instance, increased gene expressions of CD11b and IBA1 were reported in the mesenchymal subtype, suggesting a greater recruitment of macrophages/microglia compared to the proneural and classical subtypes (36). Classical GBM was also associated with greater activation of dendritic cells. Moreover, mesenchymal and proneural GBM exhibit lower populations of NK cells and CD4^+^ T cells, respectively. Strikingly, this study also reported that classical or proneural subtypes transitioning to mesenchymal upon recurrence are characterized by an increased recruitment of macrophages/microglia.

There is also a dynamic plasticity between GSCs and non-GSCs, where environmental triggers can induce stemness in non-GSCs (20, 31, 32, 51). Such triggers can include treatment with TMZ, inducing the expression of molecular markers such as CD133, SOX2, OCT4, and Nestin. These treatment-induced newly converted GSCs exhibit high tumorigenicity and infiltrative properties recapitulating the original tumor population (31). Together these studies suggest that CSCs may reflect a functional state that tumor cells can shift in and out of, depending on the environment. Fully understanding the molecular mechanisms behind these shifts in stemness will help develop effective therapies against GSCs and recurrence (20). Epigenetic factors, such as histone modification and microRNA regulation, are potential mechanisms by which cells regulate stemness (20, 51, 52). These factors strongly implicated in GSC plasticity and stemness modulation represent viable targets for therapies. In conclusion, this heterogeneity and shift between subtypes, triggered by the fluidity of the CSCs, underlie treatment resistance and disease recurrence (**Figure 1**).

## Heterogeneity of immune cells in GBM TME

**Figure 2:**
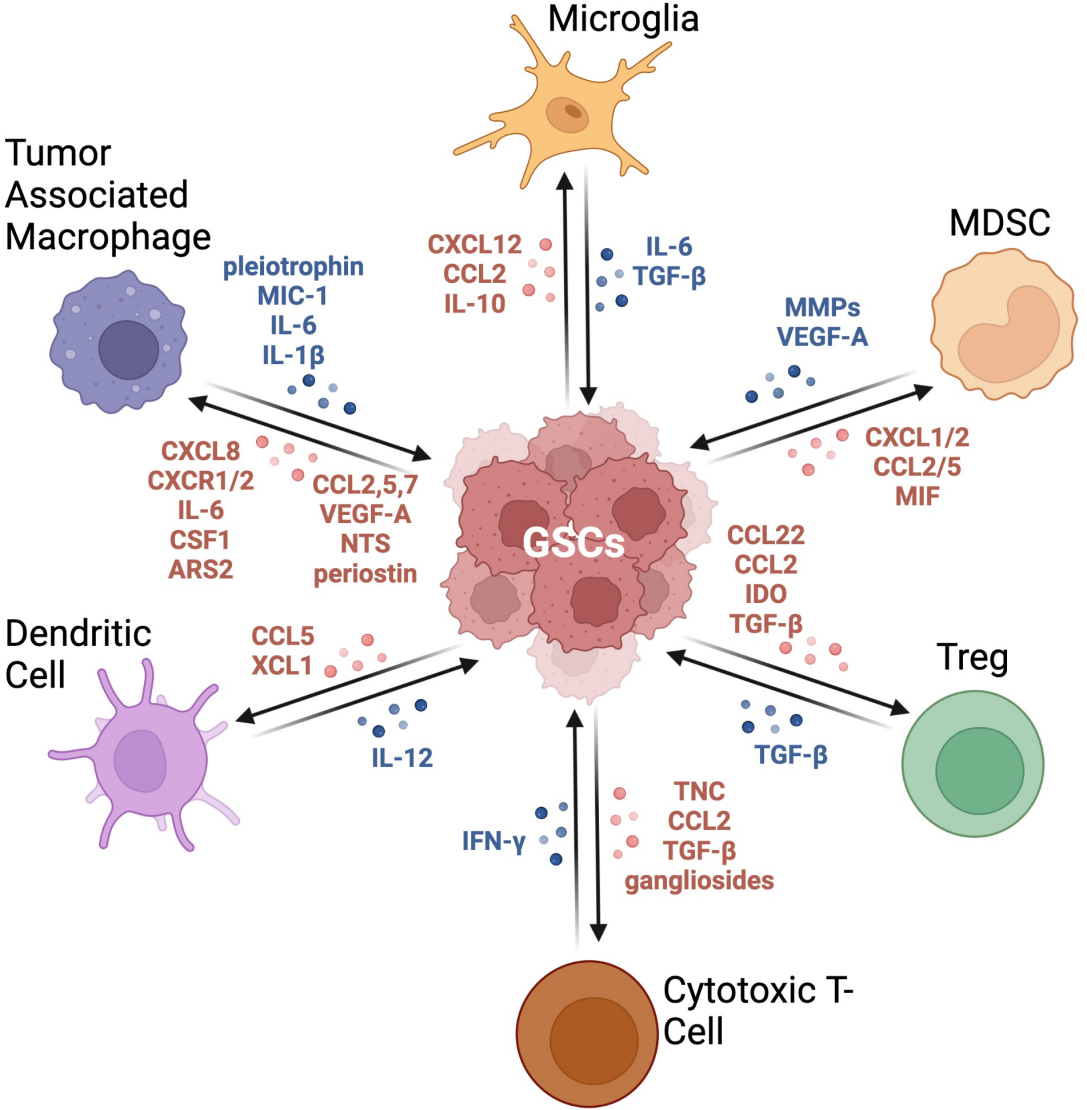
Crosstalk between GSCs and immune cells in the GBM TME. This graphic demonstrates the bidirectional relationship between GSCs and the immune compartment. These signaling pathways result in immune cells producing an immunosuppressive milieu and tumor cells becoming more tumorigenic with increased expression of stemness markers. Adapted from “Immunosuppressive Cells in the Tumor Microenvironment”, by BioRender.com (2022). Retrieved from https://app.biorender.com/biorender-templates.

The immune microenvironment of GBM plays an important role in the growth and development of tumor cells (**Figure 2**). One of the characteristics of GBM is that they are “cold” tumors, characterized by the lack of a strong immune response. Such tumors are enriched in cells that are able to limit an anti-tumor immune response by inhibiting dendritic cell and T cell migration and activities (53). The main immune cells in the GBM TME are macrophages, microglia, myeloid-derived suppressor cells (MDSCs), T-regs (T cell subset), dendritic cells (DCs), and neutrophils. These different immune cells can be distinguished within the GBM TME through various cell surface markers. Many of these markers are shared between humans and mice, however some are unique to their respective species (**Table 1**). Typically, tumor associated macrophages (TAMs), MDSCs, and T-regs are upregulated in GBM, contributing to their immunosuppressive nature, with higher populations associated with poor prognosis (75). Conversely, DCs are downregulated, further supporting immune suppression in GBM (75). B cells, especially regulatory B cells (Breg), have also been identified and described in the GBM microenvironment (76). Lee-Chang and colleagues demonstrated that GBM-associated MDSCs promote Breg function *via* transfer of PD-L1, conferring Bregs the capacity to suppress CD8^+^ T cell activation and acquisition of an effector phenotype (76).

**Table 1:** Non-exhaustive list of markers expressed by GBM-associated immune cells.

Immune Cell Type	Human	Mouse
**Microglia**	CD11b, Tmem119, P2RY12, CD45^low^, TLR4^low^ (54–58)	CD11b, Tmem119, P2RY12, CD45^low^, TLR4^high^, IFNγ receptor (54, 56, 57)
**Macrophages**	CD45^high^, CD14, CD11b M1 macrophages: HLA-DR, iNOS, M2 macrophages: CD206, CD204, CD163 (54, 57, 58)	Arg1, F4/80, Ly6G/C, CD11b M1 macrophages: pSTAT1, M2 macrophages: CD206, CD204, CD163 (58–60)
**MDSCs**	CD11b, CD33, CD15 (61–63)	CD11b, Gr1, Ly6G, Ly6C^low^ (61–63)
**Tregs**	CD4, CD39, CD127^low^, FOXP3^high^, Ki-67 (61, 64)	CD4, CD39, CD127^low^, FOXP3^high^ (61, 64)
**Dendritic cells**	cDC1: CD45, CD11c, DNGR1, CD141, XCR1 cDC2: CD1C, CD207, CD11b, CD11c, NOTCH2, SIRPA pDC: CD303, CD85k, CD304, and CD197 mo-DC: CD14, CD206, CD209, SIRPA, CD11b, CD1A (65–71)	cDC1: CD45, CD11c, MHC class II, CD8, CD24, CLEC9A cDC2: CD45, CD11c, MHC class II, CD4, SIRPa, CD11b pDC: CD45, CD11c, MHC class II, CD317, Siglec-H, Ly-6C, B220 Inflammatory DC: CD11c, MHC class II, CD64, and CD11b (62, 71, 72)
**Neutrophils**	CD11b, CD16, CD66b (73, 74)	CD11b, CD16, CD66b, Ly-6G (73, 74)

The main immune populations in the GBM TME are microglia, macrophages, MDSCs, Tregs, dendritic cells, and neutrophils. These can be distinguished based on the cell surface markers listed above and corresponding to each immune cell type.

Microglia encompass 5-20% of the total glial cell population in the adult brain, but their role in the GBM TME is still poorly understood (77). Arising from immune progenitors in the yolk sac during early embryogenesis, they play a primary role in immune surveillance and maintaining overall tissue health through phagocytosis of cellular debris and dead neurons (78). Microglia have been shown to be highly heterogeneous, plastic, and dynamic (79–81). Microglia comprise the majority of CD45^mid^ cells in human IDH mutant (IDH^mut^) glioma that exhibit smaller numbers of monocyte derived macrophages and fewer lymphocytes or neutrophils compared to human IDH wild-type (IDH^wt^) glioma (54, 82). scRNA sequencing analyses coupled with cytometry by time of flight (CyTOF) studies of microglial heterogeneity in human IDH^wt^ GBM identified nine microglia clusters (54, 83). Microglia have also been described to stimulate tumor cell infiltration. This was seen in murine glioma cells when the tumor cells without microglia had decreased and delayed tumor cell migration compared to cells with intact microglia (84). Upregulation of matrix metalloproteinases (MMPs), such as MMP2 and MMP4, by microglia plays a crucial role in enabling cancer cell migration and metastasis through the degradation of extracellular matrix (ECM) macromolecules such as collagens, laminins, and proteoglycans (85, 86). Other upregulated MMPs connected to microglial expression include MMP9 or MT1-MMP, which act through toll-like receptors and the p38 MAPK pathway (87, 88). Caponegro and colleagues demonstrated that microglia contribute to the recruitment of macrophages and regulatory T cells by releasing CCL2, both contributing to the immune escape of tumor cells (89). Post-transcriptional regulatory mechanisms modulating metabolic, inflammatory, and interferon-related pathways, regulate the microglial responses in the GBM TME (83). In multiple mouse models of glioma, microglia were found predominantly at the tumor margins promoting spatially related functions of glioma cells including proliferation, infiltration, and stemness (89–92).

The brain TME is also comprised of monocyte-derived macrophages, which make about a third of the entire tumor mass, and their abundance correlates with glioma grade (93). Key differences exist between brain tumor associated monocyte-derived macrophages and microglia with respect to ontogeny-specific transcription factors and their respective spatial distributions (94, 95). Functional and phenotypic differences were also noted between these two immune cell types (54). Tumor associated monocyte-derived macrophages are classified by their activation state, previously grouped into the classically activated M1 (anti-tumor effects) or alternatively activated M2 group (pro-tumor effects) (96). Proinflammatory cytokines such as IFN-γ and TNF-α, as well as TLR (toll-like receptor) ligands, are associated with the M1-like characteristics, while anti-inflammatory cytokines such as IL-4, IL-3, and TGF-β are associated with the M2-like characteristics (97, 98). However, this dichotomy of M1-M2 macrophages is more complex and nuanced with a broader range of macrophage phenotypes and function along the spectrum defined by the M1-M2 extremes. This was shown when human macrophages were activated with 28 different stimuli and had differing activation responses (99). Thus, rather than the dichotomous M1-M2 model, distinguishing macrophages through the expression of gene clusters, defining signatures, or inducing stimuli, may be a more accurate depiction of macrophages diversity (100). In a model generated through single cell -omics, and spanning multiple cancer types, seven groups of TAMs were described with varying signature genes, enriched pathways, and predicated function: interferon-primed TAMs (IFN-TAMs), immune regulatory TAMs (Reg-TAMs), inflammatory cytokine-enriched TAMs (Inflam-TAMs), lipid-associated TAMs (LA-TAMs), pro-angiogenic TAMs (Angio-TAMs), RTM-like TAMs (RTM-TAMs), and proliferating TAMs (Prolif-TAMs) (98). IFN-TAMs most closely resemble M1-like macrophages, but have an immunosuppressive function through tryptophan degradation and Treg recruitment (101). Reg-TAMs most closely resemble M2-like macrophages with an immunosuppressive function regulated by triggering receptor expressed on myeloid cells 2 (TREM2) with characteristics dependent on the signature genes induced in specific cancers (98, 102). Inflam-TAMs are involved in the tumor inflammatory response, aiding in recruiting and regulating immune cells to the site of inflammation (98). Angio-TAMs are involved in tumor progression, tumor cell intravasation, extravasation, and chemotherapy resistance with a large population seen in hypoxic regions of the GBM TME, and are associated with worse patient prognosis (103–105). LA-TAMs help suppress the anti-tumor immune response to promote tumor progression through the immunosuppressive lipid catabolism and inflammation-promoting lipid synthesis (106, 107). RTM-TAMs have a high level of heterogeneity and have been shown to promote tumor invasiveness through the induction of tumor cell epithelial–mesenchymal transition (EMT) and Treg recruitment in a GBM model (108). Prolif-TAMs expand through proliferation to promote tumor progression, playing an important role in tumor growth and may function as precursors to other TAM subsets (98, 109). These are not the only monocyte subtypes but can serve as means of organization and macrophage classification, with the main signature genes and markers for each subtype listed in **Table 2**. TAMs display a large range of CX3CR1 and CCR2 expression levels, suggesting that there is a frequent transformation of infiltrating monocytes into mature macrophages (128). They also enhance immune suppression and angiogenesis by expelling specific anti-inflammatory cytokines, such as TGFβ or IL-10, and angiogenic factors, such as VEGFα (97). IL-10 acts to promote tumor growth through the JAK2/STAT3 pathway (129). An activation loop is formed as STAT3 is transcribed, causing suppression of nearby immune cell activity, also leading to a reduction in IFN-γ and TNF-α in GBM, preventing all anti-tumor activity and creating a pro-immunosuppressive environment (129). GBM cells regulate the recruitment and phenotype of monocyte-derived macrophages in the TME. For instance, macrophages can vary in composition and have been shown to be influenced by mutations in the IDH1 or IDH2 genes, encoding for isocitrate dehydrogenase and resulting in the production of oncometabolite 2-hydroxyglutarate (130).

**Table 2:** Non-Exhaustive Signature Genes and Markers for Tumor-associated Macrophage (TAM) Diversity.

TAM subset	Human	Mouse
IFN-TAMs	CXCL10 (103, 110, 111), PDL1 (112, 113), ISG15 (103), CD86 and MHCII (112)	Ccl2/7/8, Cd274, Cxcl9/10/11, Ifit1/2/3, Isg15, Nos2, Rsad2, Tnfsf10 (104, 113, 114)
Reg-TAMs	ARG1, MRC1, CX3CR1, TREM2 (102, 113, 115)	Apoe, Arg1, Cx3cr1, Hmox1, Mrc1, Pf4, Spp1, Trem2, Itga4 (113, 115, 116)
Inflam-TAMs	IL1B, CXCL1/2/3/8, CCL3, and CCL3L1 (103, 114, 117–121)	Cxcl1/2/3/5/8, Ccl20, Ccl3l1, Il1rn, Il1b, G0s2 (114)
Angio-TAMs	VEGFA, SPP1, VCAN, FCN1, THBS1, STAT3 (103, 117, 119, 122, 123)	Arg1, Adam8, Bnip3, Mif, Slc2a1, Stat3 (104)
LA-TAMs	APOC1, APOE, ACP5, FABP5 (112, 117, 119–121, 124, 125)	Acp5, Apoc1, Apoe, Fabp5, Gpnmb, Lgals3 (104, 114, 125)
RTM-TAMs	LYVE1, HES1, FOLR2 (15, 110, 112, 126)	Bin1, Cst7, Hexb, Nav3, P2ry12, Sall1, Siglech, Sparc (104)
Prolif- TAMs	Ki-67, CDK1, CDC45, HMGB1 (98, 127)	Cdk1, Mki67, Stmn1, Top2a, Tubb (102, 104, 114)

Myeloid-derived suppressor cells (MDSCs) are activated neutrophils and monocytes that have immunosuppressive activity. During GBM development, the integrity of the blood brain barrier is compromised, leading to the infiltration of inflammatory monocytes, which enter the brain tissue and differentiate into MDSCs (90). This leads to an abundance of MDSCs within the GBM TME. MDSCs can be characterized into three main subtypes: granulocytic (G-MDSCs), mononuclear (M-MDSC), and early-stage (eMDSCs), each with different roles and functions (131). G-MDSCs can be separated due to their low-density properties, and eMDSCs are immature, lineage-negative cells that do not express some of the common MDSC markers such as CD15 (131, 132). M-MDSCs have the highest immunosuppressive capacity through the secretion of immunosuppressive cytokines, suppressing T cell function and promoting Treg cells through the secretion of TGF-β and IL-10 (133, 134). G-MDSCs can also play a role in T cell immunosuppression through increased expression of S100A8/9 and arginase, and the production of reactive oxygen species (ROS) (135, 136). One study found that MDSCs had significantly impaired CD4^+^ T cell memory functions in GBM patients (61). They detected a strong association between G-MDSCs and CD4^+^ effector memory T cells, along with upregulated PD-L1 expression associated with driving T cell exhaustion in the TME (61). The CD74 receptor is also overexpressed in MDSCs as a macrophage migration inhibitory factor (MIF) receptor, especially in the M-MDSC subtype (137). CD74 mediates the MIF signal transduction and leads to the recruitment of CD44 and downstream Src/MAPK signaling, which promotes oncogenesis (138). Recent work reported an increased level of circulating M-MDSCs in the blood of GBM patients compared to low grade glioma (136, 139). In addition, these studies identified that an increase in MDSCs infiltrating the GBM microenvironment correlated with poor prognosis.

Tumor-associated neutrophils (TAN) are also observed in GBM (54). Neutrophils have also been seen to promote the progression and proliferation of GSCs through upregulation of S100 protein-dependent mechanisms (140), as well as induce immunosuppression of other immune cells through the production of arginase 1, involved in hydrolysis of L-arginine to produce urea and L-ornithine (141).

T cells represent a small percentage of the immune cells within the GBM TME; however, T cell dysfunction still plays an important role in tumor development (142). Among the populations of CD8 cytotoxic T cells and CD4 helper T cells, regulatory T cells (Tregs) are a critical subpopulation of CD4^+^ T cells involved in preventing autoimmunity and having a greater association with GBM prognosis (143). Tregs can be split into two groups: the thymus derived Tregs, which express high levels of FoxP3 and develop after antigen presentation by thymic epithelial cells, and the peripherally induced Tregs, which differentiate in the periphery after antigen presentation and recognition by CD4^+^ T cells (143). The strong immunosuppressive microenvironment described in GBM is associated with an abundance of Tregs and worse prognosis (75). GSCs attract Tregs by secreting CCL22, CCL2, and TGF-beta chemokines (**Figure 2**) which bind to CCR4, a target commonly expressed on Tregs. High indoleamine 2,3-dioxygenase 1 (IDO1) levels are also correlated with a decreased GBM patient survival, and it was shown that Tregs facilitate IDO1 immunosuppression, leading to decreased activities of CD8^+^ effector T cells and overall T cell immune response (144). Additionally, Nrp1 in Tregs has an important function in suppressing the anti-tumor immune response (145). The interaction of Nrp1 with the ligand, semaphorin 4a, stabilizes the Treg phenotype, and the loss of Nrp1 leads to the loss of this immunosuppressive nature (145). Interestingly, in human IDH^mut^ glioma, 2-hydroxyglutarate released by tumor cells can be transferred to T cells, where it interferes with calcium-dependent transcriptional activity of nuclear factor of activated T cells (NFAT), polyamine biosynthesis, and ATP-dependent TCR signaling, resulting in the suppression of T cell activity (146). This illustrates how tumor metabolic activities can shape and modulate the tumor immune microenvironment.

The dendritic cell (DC) population is very limited in the GBM TME due to their low intra-tumor infiltration, further contributing to GBM evasion of immune surveillance (75). However, some DC subsets have been identified in GBM such as conventional DC1 (cDC1), cDC2, migratory DCs, pre-DCs, and plasmacytoid DCs (pDCs). Their presence suggests a potential for an anti-tumor immune response that could be exploited in the context of immunotherapies. Recruitment of DCs could be stimulated by CCL5 and XCL1, which in turn would produce cytokines such as IL-12, promoting the anti-tumor activity of T cells and NK cells (147).

## Geospatial distribution of GSCs and association with the immune landscape

The utilization of spatial profiling in providing an architectural context to tumors is being increasingly recognized as a critical method to understand the fundamental mechanisms driving diseases and to develop novel therapies. As mentioned above, GBM subtypes, GSC populations, and their corresponding tumor microenvironments are spatially defined within a single tumor (35). Specific GBM niches have been classified as perivascular, perinecrotic, or hypoxic and invasive (148). These are areas where GSCs can interact specifically with the microenvironment, regulating metabolism, supporting survival and growth, and affecting immune surveillance (91). These spatially resolved niches can shape and maintain specific GSC populations. The perivascular niche is characterized by contact between tumor cells and vasculature, specifically endothelial cells (148, 149). This contact supports tumor growth, diffusion, and treatment resistance. Lineage tracing experiments revealed that GSCs can differentiate into pericytes to promote angiogenesis (150). Another study showed specifically that CD133^+^ GSCs are able to differentiate into endothelial cells (151). Furthermore, tumor growth is also supported by the angiogenic and immunosuppressive properties of monocytes, neutrophils, and MDSCs that are found specifically in the perivascular niche (91, 152, 153). Perinecrotic/hypoxic niches were also associated with enhanced stemness of tumor cells by promoting self-renewal, proliferation, and survival (91, 148, 154–156). These niches are also correlated with increased PD-L1 expression in GSCs, further contributing to immune evasion from T cells, as previously discussed (76, 157). The invasive niche is characterized by GBM cells invading normal tissue and building vessels through the release of factors such as angiopoietin 1 and 2, and VEGF. Microglia and macrophages were found to be recruited to tumor invasive edges, where they also promote immune suppression, enhance tumor cell stemness, and lead to treatment resistance (158). These studies support the specific relationship between GSCs and immune cells in different spatially resolved niches, and that the cellular contexture in the tumor immune microenvironment (TIME) plays a pivotal role for GSCs. Notably, these different regions have been shown to respond differently to treatments and therefore modulating disease presentation and progression. For instance, interactions of tumor cells with endothelial cells reduces their sensitivity to radiation (43, 159). Garcia-Barros and colleagues demonstrated that tumors grown with endothelial cells that are resistant to radiation-induced apoptotic death exhibited reduced radiation damage and enhanced tumor growth when exposed to irradiation in comparison to tumors grown with normal endothelial cells (160). Additionally, the hypoxic niches have shown to not only increase self-renewal, but also the expression of MGMT in GSCs, further contributing to resistance to alkylating chemotherapies such as TMZ (159, 161). These studies demonstrate a direct supportive function of the TME in the maintenance of tumor cells and GSCs. Piccirillo and colleagues reported differential GSC signatures between spatially distinct tumor regions (core vs periphery), further supporting a spatialized distribution of GSCs (21). The study also revealed that while these core and periphery GSCs shared a common ancestry, they both differ in tumorigenic potential, growth kinetics, and phenotype, illustrating the functional range of stemness present within the same tumor (162). Yang et al., used a combination of optical tissue clearing methods (CUBIC and iDISCO^+^) and deep tissue imaging with two-photon microscopy to also reveal intratumoral spatial heterogeneity in GBM in terms of GSC marker expression, microvasculature, and immune contexture (163).

Significant efforts are currently underway to study the TIME by not only investigating its molecular features and cellular compositions, but also characterizing its spatial architecture. Schaettler and colleagues demonstrated that spatial diversity in brain tumors is also recapitulated in the distribution of immune cells (164). This study reported a comprehensive immunogenomic profiling of multiple spatially distinct areas from a large cohort of GBM patients. The authors adopted specific immune deconvolution methods to resolve immune cell populations from transcriptional data to characterize multiple tumor regions (165, 166). Substantial inter-tumoral variation was observed, specifically among CD8^+^ T cell and cytotoxic T cell scores. Together, this demonstrates that the TIME within GBM is also spatially resolved.

## Metabolic heterogeneity in GSCs

**Figure 3:**
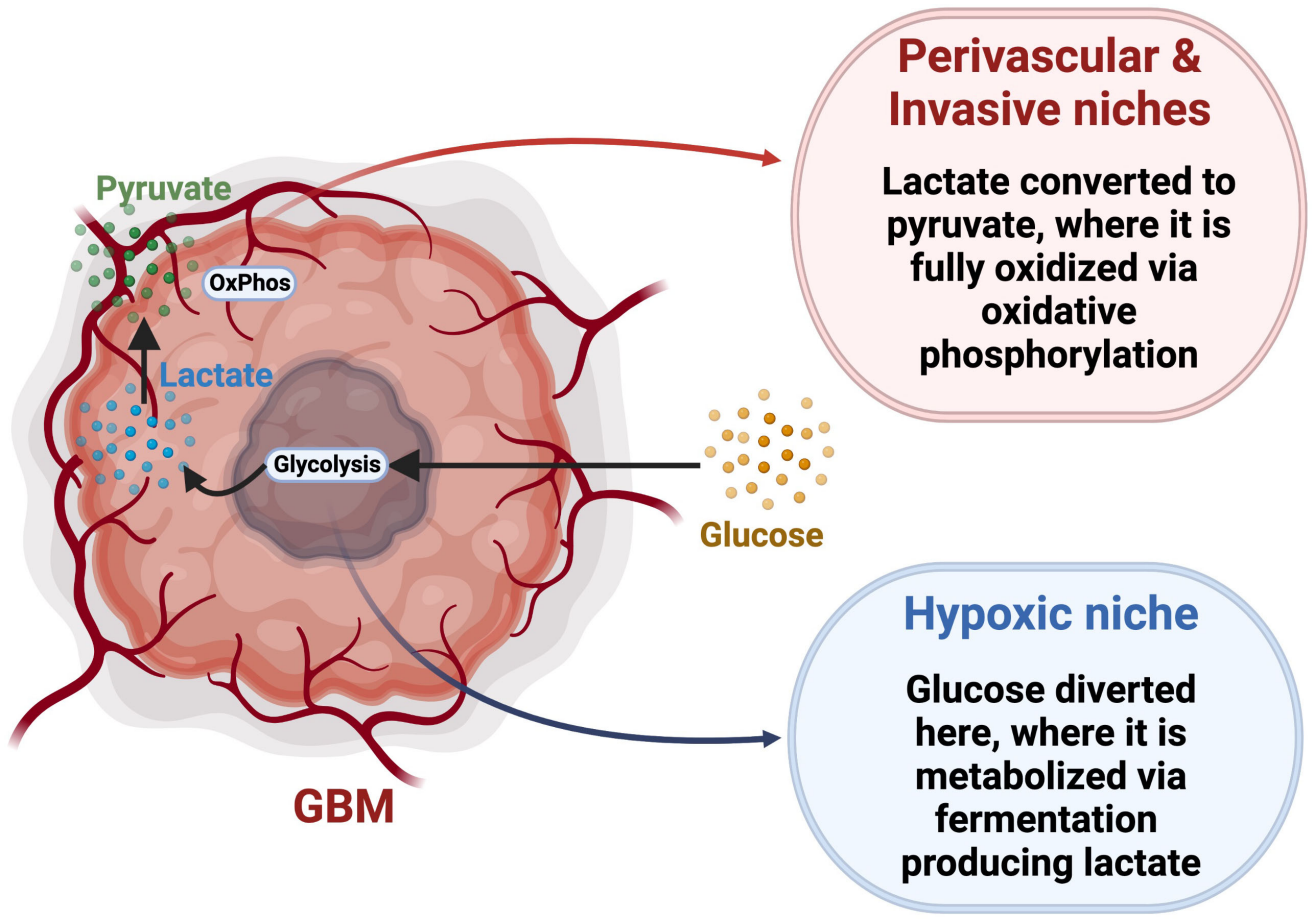
Reverse Warburg Effect hypothesis. This model proposes a specific metabolic interplay between glycolytic and oxidative tumor niches *via* trafficking and conversion of glucose, lactate and pyruvate. Adapted from “Cancer Metabolism”, by BioRender.com (2022). Retrieved from https://app.biorender.com/biorender-templates.

The Warburg hypothesis proposes that cancer cells exhibit impaired mitochondrial function and utilize primarily glycolysis *via* aerobic fermentation, generating lactate and ATP, bypassing OxPhos even in an abundance of oxygen (167, 168). Most GBM tumor cells follow the Warburg hypothesis, as evidenced by the overexpression of pyruvate kinase M2 (PKM2) and lactate dehydrogenase, enzymes that plays a key role in glycolysis by directing glucose metabolism towards the production of lactate (169, 170). Higher expression of glycolytic genes in GBM results in a more aggressive and lethal phenotype with increased lactate production facilitating biosynthesis of lipids, nucleotides, and macromolecule production, overcoming the reduced availability of nutrients in the TME and supporting tumor cell survival and proliferation (171–173). Also, GBM cells compete with immune cells for glucose, as activated T cells and NK cells, macrophages, and neutrophils have all been reported to exhibit a metabolic shift towards aerobic glycolysis within the TME, especially during hypoxic conditions, consequently increasing their need for the nutrient (174, 175). The GBM TME is therefore characterized by high demand for glucose but with limited availability, creating a strong intercellular metabolic competition (176). Multiple studies reported that GSCs show metabolic adaptability and are able to utilize multiple nutritional substrates to satisfy their metabolic needs, conferring an advantage over less-plastic tumor cells or non-malignant cells (168, 177). Accordingly, this provides an advantage over differentiated glioma cells with more defined metabolic dependencies, and allows them to resist and adapt to metabolic pressures. This metabolic adaptability of GSCs would suggest tolerance to treatments targeting a single specific metabolic pathway (178). For example, GSCs with a slow-cycling phenotype were found to be more resistant to glucose deprivation than differentiated tumor cells (14). To overcome metabolic pressure related to glucose restriction, GSCs metabolize lipids to meet their energy demands (14, 179). Wang and colleagues found that, in breast cancer, when fatty acid β-oxidation (FOA) is inhibited, breast cancer stem cells become less resistant to treatment, demonstrating their dependence on fatty acids (180). Concurrently, inhibiting fatty acid acyl-CoA synthetase VL3 (ACSVL3), an important enzyme in the lipid metabolic cascade that facilitates the synthesis of phosphatidic acid, decreases expression of CD133 and SOX2 in glioma cells (181). When mice were fed a high fat diet, the intracranial accumulation of saturated lipids caused GBM to become more lethal, with an enrichment of GSCs (182). Even in the absence of exogenous lipids, GSCs can synthesize cholesterol and fatty acids from acetyl-CoA, which allows for long term fuel storage (183). In addition to PKM2, GSCs express PKM1, allowing them to engage in glycolysis through both fermentation and oxidation (169). GSCs isolated by sphere formation assay were also found to produce more ATP than differentiated cells while metabolizing less glucose and producing less lactate (178), further suggesting their engagement in OxPhos for ATP production. While aerobic glycolysis may confer an advantage to rapidly proliferating cells, GSCs, which can be slow-cycling, rely more heavily on OxPhos for efficient energy synthesis to support their infiltrative phenotype (14, 168). However, CD133^+^ GSCs in the hypoxic core have been shown to overexpress GLUT3 (16), illustrating a variety of metabolic phenotypes amongst GSC populations. It was also proposed that glucose can be directed towards hypoxic niches, where it is metabolized through homolactic fermentation generating lactate (**Figure 3**) (184, 185). This lactate is then transported to the perivascular and invasive niches, where it is converted into pyruvate by lactate dehydrogenase B and fully metabolized using OxPhos (184, 185). This demonstrates the complex metabolic interplay between GSC niches, supporting the idea that GSCs are capable of switching between different metabolic strategies based on nutrient availability. This concept of the reverse Warburg effect has not been extensively accepted, and more research is required to fully elucidate the complex relationship between these distinct niches in the TME. Interestingly, GSCs are not a homogeneous population, but rather a mosaic of different genetic lineages which share certain stem-cell properties (5, 12). Each lineage, characterized by specific metabolic profile and stem cell marker expression, may vary with microenvironmental variations, including metabolic changes (10). This reveals the challenges of finding a treatment universally targeting GSCs, and suggests the requirement of combinatorial strategies to encompass GSC diversity and adaptability.

## Metabolic heterogeneity of immune cells in GBM TME

The immune cells in the TME are programmed to rapidly respond to tissue-derived or environmental stimuli, with their function tied to metabolic signals (186). This includes differential activation states, cellular reprogramming, and stimulated expression (186). It has been shown that heterogeneity between the pro-inflammatory and anti-inflammatory macrophage phenotypes results in different regulation of lipid handling and metabolism (187). The GBM microenvironment is marked by an increase in anti-inflammatory macrophages and a decrease in pro-inflammatory macrophages (188–192). The pro-inflammatory phenotypes have been described to primarily utilize Warburg metabolism, further fueled by the highly active u-PFK2 isoform of phosphofructokinase-2 that promotes glycolysis (193), while anti-inflammatory macrophages generate energy through enhanced mitochondrial OxPhos (187, 194). Anti-inflammatory macrophage metabolism is driven by engulfed lipids that are oxidized by FAO and facilitated by the fatty acid sensor, PPARγ (186). FAO is linked to pro-tumorigenic macrophage state, and acts as a source of ATP in the mitochondrial matrix during low glucose availability, driven by IL-4 and IL-13 (195). Lipid metabolism in macrophages is linked to tissue homeostasis and phagocytosis, and depends on four cellular processes—lipid uptake, efflux, biogenesis, and conversion into other metabolites or lipid intermediates (186). Lipids are recycled from necrotic and apoptotic cells through uptake by macrophages, and then generated into free fatty acids that can be utilized by other cells (196). Macrophages scavenge lipids from the TME through CD36 endocytosis induced by IL-4 (196, 197). Excess fatty acids are stored in lipid storage organelles called lipid droplets, which can be later converted back into fatty acids for energy production, and play critical roles in lipid trafficking, lipid homeostasis, and metabolism (198). These recycled lipids can also be transported to and utilized by surrounding cells. Our group showed that slow-cycling GSCs upregulate signaling pathways controlling and enhancing lipid metabolism. Recruiting macrophages and establishing a metabolic coupling with these cells may be a strategy by which the slow-cycling GSCs to support their metabolic needs through transfer of critical signals and nutrients, such as fatty acids (14, 168). Further study will be required to fully understand the nature of this potential hetero-cellular metabolic symbiosis and identify strategies to disrupt it for therapeutic purposes. Similar to cancer cells, T cell functions are metabolically regulated, with different subsets of T cells undergoing various metabolic reprogramming and energy usage along the differentiation spectrum. Effector T cells engage anabolic metabolism and quiescent or memory T cells undergo catabolic metabolism (199–201). Naïve T cells rely on fatty acid oxidation with a lower glycolytic rate compared to the other subtypes, whereas conventional activated T cells preferentially utilize glycolysis, resulting in lactate production (199, 201). However, variable levels and inconsistent availability of glucose in the GBM TME can lead to impaired activation of cytotoxic T cells (199). In this context, Tregs present an advantage by being able to oxidize glucose and fatty acids through the upregulation of carnitine palmitoyltransferase 1a (CPT1a), an enzyme regulating the metabolism of acyl groups (202). Tregs express the transcription factor, Foxp3, which is involved in lineage development and metabolic function (203). It has been shown that the induction of Foxp3 expression is sufficient for the shift from aerobic glycolysis to OxPhos, conferring resistance to low-glucose and high-lactate exposure, as experienced in the GBM TME (204). Together, these processes contribute to the overall immunosuppressive nature of the GBM TME.

As key components of the TME, MDSCs also undergo metabolic reprogramming regulating inflammation programs, controlled by oxygen, nutrient, and metabolite levels (174). The metabolic activities of MDSCs contribute to depleting amino acids (e.g., tryptophan, L-arginine, and cysteine) that are crucial for T cell function and activation, leading to their suppression (205–208). MDSCs are capable of both glycolysis and fatty acid metabolism, regulated by different signaling complexes, PPARγ and mTOR, that can sense extracellular glucose and metabolite status (209). PPARγ supports the expression and synthesis of pro-inflammatory cytokines, promoting MDSC expansion and immunosuppression (210). Deletion of mTOR complexes led to reduced MDSC differentiation and immunosuppressive function (207). The hypoxic conditions of the TME stimulate the immunosuppressive function of MDSCs and lead to increased uptake of extracellular nutrients required for glycolysis and fatty acid oxidation (211). Additionally, lactate produced by tumor cells can be taken up by MDSCs and used to support their metabolism and immunosuppressive activities (211). Using PET tracers, Reinfedl and colleagues investigated the access and incorporation of nutrients, such as glucose and glutamine, by multiple cell subsets in the TME (212). The study reveals great competition for limited nutrients between cancer cells and immune cells. Specifically, the authors reported that myeloid cells exhibit the greatest ability to uptake and metabolize glucose and tumor cells show the highest uptake of glutamine, both contributing to nutrient deprivation for T cells, impairing their survival, maintenance, and activity. This study showed that T cells compete for essential nutrients not only against tumor cells but also against other immune cells such as myeloid cells.

## GSC-immune cell interactions in the TME

The GBM TME hosts an extremely complex network of tumor and immune cells. A hallmark of GBM is its ability to generate an immunosuppressive milieu supported by the bidirectional crosstalk between GSCs and immune infiltrates, subverting the effects of cytotoxic T cells while simultaneously reprogramming other immune cells to foster a protumorigenic microenvironment (213). This property of GSCs allows for their survival of standard-of-care treatments, seeding disease recurrence. This section will present potential mechanisms by which GSCs can drive disease progression *via* specific interactions with the different immune cells composing the TME (**Figure 2**).

### Tumor associated macrophages (TAMs)

TAMs play a key role in the establishment of the immunosuppressive characteristics of the GBM TME. It was shown that by overexpressing the extracellular matrix protein periostin, CD133^+^ GSCs stimulate the recruitment of TAMs to the TME (188). GSCs were also shown to secrete factors such as CSF1, CCL2, CCL5, CCL7, VEGF-A, and NTS, also contributing to the recruitment of immune suppressive macrophages (214, 215). Macrophages are also attracted by GSC secretion of CXCL8 and CXCR1/2 chemokines (216). Not only do GSCs recruit TAMs, but they also play a role in their M2-like polarization. GSCs express arsenite-resistance protein 2 (ARS2), which activates the transcription of the MGLL gene to produce monoacylglycerol lipase (MAGL), inducing TAMs to adopt a pro-tumorigenic and immunosuppressive phenotype (217). M2 macrophages release cytokines including IL-10, TGF-b, and IL-23, which impair cytotoxic T cell function (214). CD133^+^ GSCs were shown to inhibit the phagocytosis capability of macrophages through the secretion of macrophage inhibitory cytokine 1 (MIC-1) (218). Tao et al., further demonstrated the interplay between GSCs and macrophages by showing that Wnt-induced signaling protein 1 (WISP1) expressed by GSCs promotes their maintenance by autocrine mechanism regulated by a6b1-Akt signaling and supports the survival of tumor supportive macrophages (219). Interestingly, TAMs can also induce the stem cell phenotype in GBM tumor cells by releasing mediators like IL-6 and IL-1β, or through juxtacrine signaling (15, 220). Shi and colleagues reported that TAMs secrete high levels of pleiotrophin, thereby increasing GSC maintenance and tumorigenic potential by promoting PTPRZ1 signaling (189). Together, this suggests a strong mutualistic relationship between GSCs and TAMs.

### Microglia

Microglia are intrinsically antitumorigenic; however, exposure to tumor-generated factors, such as IL-10, induces an immunosuppressive phenotype (221, 222). Microglia from healthy human brains exposed to GSCs expressing Nestin, SOX2, Musashi-1, CD133, and inhibitor of differentiation (ID4) reduced GSC sphere forming ability, but microglia derived from resected human GBM conferred an increase in their sphere forming ability (221). This shows that microglia found in the GBM TME have a modified phenotype that promotes tumor growth and survival. GSCs have been found to induce the mTOR pathway in microglia, spurring their growth within the TME (223). Microglia are major contributors of TGF-β, which amplifies immunosuppression in the GBM TME by blocking T cell activation and proliferation, inhibiting the activation of NK cells, down regulating IL-2 production, and promoting Tregs (75). TGF-β promotes tumor cell invasion through changes in ECM components, enhanced expression of subunits of α2,5, β3 integrin, and upregulated MMP-2, 9, and MT1-MMP (224). Microglia can also regulate stemness *via* the IL-6 pathways, which in turn stimulates M2 macrophage recruitment by GSCs by release of periostin (188). Chen and colleagues recently reported that circadian locomotor output cycles kaput (CLOCK) is amplified in GBM and stimulates GSC maintenance and drive immunosuppression (225, 226). These studies revealed that this circadian regulator upregulates LGMC in GSCs through activation of the HIF1a pathway, which in turn promotes the infiltration of microglia and their polarization toward an immunosuppressive phenotype.

### MDSCs

As mentioned above, MDSCs also play a critical immunosuppressive role in GBM. Patients expressing higher MDSC gene signatures correlated with worse prognosis (218). Through geospatial analyses of human GBM tumors, MDSCs were found in close proximity to CD133^+^ and SOX2^+^ GSCs; these GSCs were found to secrete the cytokine macrophage migration inhibitory factor (MIF) to recruit MDSCs (218), as well as chemokine secretion of CCL2 and CCL5 (15). Gliomas also overexpress chemokine ligands 1 and 2 (CXCL1 and CXCL2, respectively), promoting the recruitment of MDSCs from the bone marrow towards the tumor (227). A study of breast cancer found that secretion of interleukin-6 (IL-6) by tumor cells can recruit and induce an immunosuppressive phenotype in MDSCs (131). Furthermore, MDSCs show an inhibitory effect on CD8^+^ cytotoxic T cells through the production of reactive oxygen species (228). MDSCs also promote angiogenesis and tumor growth in GBM by the secretion of a variety of matrix metalloproteinases (MMPs) and VEGF-A (205). Preventing cellular signaling between MDSCs and GSCs may prove to be a beneficial therapeutic strategy.

### Regulatory T lymphocytes (Tregs)

Chang et al., reported that TAMs in glioma produce CCL2, which is essential for the recruitment of CCR4 expressing Treg (229). The authors also showed that in the absence of CCL2, Tregs failed to accumulate in the GBM TME and that CCR4-deficient mice were defective in glioma accumulation. Considering the ability of GSCs to recruit TAMs, this study suggests an indirect relationship between Tregs and GSCs. Expression of indoleamine 2,3-dioxygenase (IDO) by Nestin^+^ and SOX2^+^ GSCs has also been correlated with Treg recruitment (230). Interestingly, resected GBM tumors with IDO deficiency exhibited reduced Treg recruitment (231). Conversely, Treg secretion of TGF-β promotes a stem-like phenotype in GBM (232). Liu and colleagues demonstrated that TGF-β stimulated the expression of the cancer stem cell-related gene core CD133, SOX2, NESTIN, MUSASHI1 and ALDH1A regulated by NF-κB–IL6–STAT3 signaling pathway (232). These studies demonstrate that the relationship between Tregs and GSCs is also bidirectional.

### Cytotoxic T lymphocytes (CTLs)

Therapeutic modalities able to enhance CTL invasion and activity in the TME could lead to increased survival in GBM patients (233). However, GSCs represent a great obstacle to this strategy due to their strong ability to impair T cell function. This can occur through the expression of CCL2 and TGF-β, and the release of inhibitory factors, including gangliosides, which directly limit the functionality of CTLs (15, 234, 235). A study reported that tenascin-C (TNC) produced by GSCs inhibits T cell proliferation and activity *via* interaction with integrins, resulting in reduced mTOR signaling (236). Notably, TNC inhibitory effect on T cell activity is mediated by exosomes trafficking. One aforementioned mechanism of enhanced glucose uptake by CD133^+^ GSCs in the hypoxic core is the overexpression of GLUT3, which allows for an increase in glycolytic flux (16). This metabolic specificity of GSCs has two-fold implications for CTL activities and include deviating this essential nutrient from T cells and secreting lactate, which acts as a strong immunosuppressant (183). Together, these GSC-imposed metabolic stresses strongly contribute to the lack of CTL mediated anti-tumor immunity in GBM.

The negative associations between cancer stemness and anticancer immunity are increasingly recognized with cancer stem cell programs representing fundamental processes in disease evolution but also providing potential mechanistic understanding of the connections between intratumoral heterogeneity, antigenicity, immune suppression, and poor disease outcome (237). Experimental reports indicated that resistance to immune-mediated targeting represent an intrinsic property of CSCs and quiescent adult stem cells (238, 239). Using gene-expression-based metrics, one report identified a 109-gene signature recapitulating stemness in both malignant and nonmalignant cells and evaluated the relationship of the signature with anti-tumor immunity. The authors used ssGSEA based tools and were able to infer the cellular content of the TME and predict immune infiltration and disease projection (237, 240). However, the complexity and heterogeneity present in GSC populations render it very challenging to predict the immune contexture and response. Unfortunately, our knowledge of the specific immunoediting properties of the different lineages of GSCs is limited, and therefore requires further investigation. Integrating mechanistic insight from GSC biology, tumor immunology, and their connection may therefore help identify vulnerabilities to be exploited to boost immune surveillance and engender an efficient anti-cancer immunity in the context of GBM.

## Targeting tumor-immune interactions

Disrupting the symbiosis between tumor cells and immune cells holds great promise in the quest to treat GBM. Multiple strategies may be applied and would include inhibiting the direct cell-cell interactions or neutralizing secreted and circulating factors supporting both GSCs and immune cells.

Considering the crosstalk between GSCs and TAMs, targeting the key pathways of their connection has the potential to inhibit their properties and functions and to impair disease progression. A promising approach to achieve this goal could be the blockade of the CD47-SIRPa pathway. The binding of CD47, expressed by CSCs, with SIRPa present on TAMs, results in the suppression of CSC phagocytosis by TAMs and in preserving their function (241, 242). Therefore, inhibiting this binding represents an appealing strategy for decreasing tumor burden with more efficient targeting of CSCs through enhancing phagocytosis activities of macrophages. Multiple clinical trials (e.g., NCT02216409, NCT03512340, NCT02367196, NCT03717103, NCT02663518, NCT03013218) investigating the effect of targeting this pathway using monoclonal CD47-targeting antibodies (IBI188, Hu5F9G4, CC-90002, and SFR231) or small molecule inhibitors (ALX148, TTI-621) have been completed or are currently recruiting. Moreover, neutralizing GSC-secreted factors such as periostin was shown to impair GSC-TAM interaction and increase survival in murine models (188). In addition to targeting receptors or blocking the release of secreted factors, another potential strategy is the trapping of ligands in the TME. A study by Wei and colleagues reporting the use of a 4-1BB-osteopontin bispecific aptamer to trap and inhibit osteopontin resulted in significant activation of anti-tumor immunity and improved outcome in murine models of GBM (243). It was shown that TAMs recruitment and immunosuppressive polarization is promoted by ROBO receptor signaling that is activated by GBM-secreted peptide SLIT2 (244). The use of Robo1Fc efficiently trapped SLIT2 ligand and also resulted in strong anti-tumor reactivity in murine GBM (244).

Given the profound role of MDSCs and their interaction with GSCs in GBM, inhibition strategies to disturb their crosstalk are also considered. Specifically, Qiu et al., reported that MDSC differentiation is induced by exosome-packaged miR-1246 released by GBM cells and that restricting the expression of miR-1246 and exosomal packaging with 2-methoxyestradiol repressed MDSC tumor infiltration and delayed GBM tumor growth (245). Notwithstanding, due to the great heterogeneity of phenotypes and morphologies of MDSCs, it remains challenging to develop MDSC-targeted treatments.

CSCs express higher level of PD-L1 than non-CSCs, including in GBM, which in turn creates a positive feedback loop further increasing the stemness in tumor cells and inhibiting cytotoxic T cell activity *via* PD1/PD-L1 signaling (246–248). This suggests the potential of using immune checkpoint inhibitors, such as anti-PD1 treatment, to target this CSC-T cell interaction and to improve anti-tumor activity. However, even though such an approach has led to remarkable results, the majority of patients do not respond as efficacy is mainly dependent on the nature and composition of the TME and the genetic makeup of the tumor. We are witnessing a growing number of clinical investigations of the effect of immune checkpoint inhibitors in GBM patients using modalities such as anti-LAG-3 in combination with anti-PD-1 (NCT02658981), anti-PD1 combined with anti-TIGIT (NCT04656535), and anti-CD39 (NCT04306900), however significant positive results are still awaited.

In light of the critical role of the tumor-immune symbiosis in regulating GSC activities and in controlling anti-tumor immunity in GBM, targeting this crosstalk not only has the potential to disrupt GSC activities but also to remodel the TIME to make it more amenable and responsive to immunotherapies. However, due to the complexity of these cellular networks and connections exhibiting redundancy and multiple compensatory mechanisms, combinatorial therapeutic paradigms may be required to achieve efficient TME remodeling leading to greater outcomes.

## Metabolic targeting of the GBM-immune crosstalk

Immune evasion and metabolic reprogramming are now well recognized hallmarks of cancer and considered to be functionally linked (**Figure 1**). Consequently, targeting pathways underlying the metabolic interplay between tumor cells, especially GSCs and immune cells, has therapeutic potential with the ability to condition the TME to be more permissive and responsive to immunotherapies.

GBM displays a high degree of hypoxia, which mediates stemness and induces T cell exhaustion through mitochondrial fragmentation (249, 250). The main factors activated in response to hypoxia are hypoxia-inducible factors (HIFs), with HIF2a being specifically upregulated in GBM and colocalized with GSC markers such as CD133 and OLIG2 (156, 251). Countering hypoxia-related signaling to target GSCs and stimulate anti-tumor immunity is a strategy currently being explored. A study recently investigated the effect of a HIF2a inhibitor (PT2385) in combination with nivolumab; however, the results encouraged the investigation of a second-generation of HIF2a inhibitor (252).

As discussed above, the metabolic switch observed in GBM leads to increased glycolysis in tumor cells and impacts the tumor microenvironment, which in turn acts as a major barrier for successful targeting of cancer by anti-tumor immune cells like T cells. Overexpression of glucose transporters and upregulation of the PI3k-Akt-mTOR signaling support the energy demand associated with this metabolic reprogramming and blockade of this pathway using an Akt inhibitor has shown to decrease glioma growth (16, 253–255). The tremendous increase in tumor glucose consumption imposes a great metabolic pressure on T cells, which experience glucose restriction. Strategies to enhance glycolytic flux and activities in T cells may prove efficient to improve T cell function and prevent or delay T cell exhaustion.

The increased glycolytic activity of GBM cells results in the massive production and secretion of lactate into the tumor microenvironment. Lactate acts as an oncometabolite and serves as a potent inhibitory regulator of T cells (256, 257). For many years, lactate was seen as just a metabolic waste product, but recent studies have revealed new roles of lactate in the TME as part of metabolic fuel or a signaling molecule regulating angiogenesis, invasion, resistance to treatments, and immunological escape. Consequently, lactate trapping may represent a viable strategy to overcome this metabolically driven tumor-imposed immunosuppression. He et al., recently reported on a new lactate “nanofactory” based on the nano-packing of lactate oxidase (LOX) by cationic polyethyleneimine (PEI) coupled with copper ions (258). This study demonstrated the ability of this system to actively trap lactate and promote its degradation, resulting in the formation of anti-tumor ROS, mediating an immunological response in a breast cancer model.

A study by Villa and colleagues reported that GBM cells are highly dependent on cholesterol for survival (259). The enrichment of the GBM TME in myeloid derived immunosuppressive cells, which have been described to exhibit specific metabolic characteristics specializing them in lipid and cholesterol transport and exchange, suggests a potential metabolic support to GBM cells provided by immune cells through the form of lipid and cholesterol transfer (196, 260, 261). Therefore, perturbation of cholesterol and lipid trafficking may have the potential to impair this metabolic crosstalk. Considering the mechanism of action of statin drugs (targeting lipid/cholesterol pathways), their widespread clinical use, well-characterized safety profile, and documented ability to inhibit the immunosuppressive function of TAMs, their utilization in the context of GBM may be considered (262, 263). Retrospective studies analyzing the effect of statins in GBM patients have not provided conclusive positive results; however, the metabolic and immunomodulatory function of statins may not be sufficient to enable efficient anti-tumor response and may require a combinatorial approach with immune checkpoint inhibitors (ICI) to achieve a significant and durable therapeutic effect. Indeed, the concomitant use of statins during ICI treatments has been correlated with improved survival in cancer patients (264–267).

## Emerging technologies

This review provides some examples illustrating the complex crosstalks that GSCs establish with tumor infiltrating immune cells. Moving forward, the systematic use of a combination of advanced technologies, such as single cell RNA sequencing, spatial profiling, metabolomics, CyTOF, and machine learning, will provide more comprehensive platforms for a deeper understanding of the composition and dynamics of the TME and evaluation of novel therapeutic modalities for a more effective translation of preclinical findings into the clinic.

For example, Bulk RNA sequencing has been instrumental in advancing our knowledge in the genetic drivers of cancer, however, this platform has inherent limitations that restrict a deeper characterization of the TME and TIME and understanding of the function and phenotype of individual cell types. Conversely, high-dimensional technologies, such as single cell RNA sequencing, overcome many limitations related to conventional profiling techniques and are helping propel forward the field of cancer research by facilitating breakthroughs in dissecting the phenotypic and functional heterogeneity among single cells, understanding the overall biology of cancers, discovering biomarkers, perfecting diagnosis, and measuring and predicting response to treatments.

Spatial analysis of disease mechanisms is currently gaining tremendous momentum and commercialization of these technologies has been critical for boosting the democratization of their implementation. Spatial transcriptomics and proteomics will allow the generation of high-dimensional spatially resolved atlases of diseases such as cancers. Multiple spatial techniques can be applied and can include gene and protein expression analyses on microdissected tissues, *in situ* sequencing, *in situ* hybridization or capturing, and computational reconstruction of spatial data. There are, however, limitations of image acquisition and processing for entire tissues or organs that exceed capabilities of most current methodologies, which requires region sampling by selecting areas of interest. This selection represents another limitation in terms of unbiased identification of locations of interest in the architectural heterogeneity of tumors. Possible solutions may come from improved computing abilities and the advancement and utilization of machine learning algorithms, which may facilitate the next logical generation of spatial profiling techniques that will involve spatial single-cell ‘omics, followed by the ultimate resolution of spatiotemporal 3D single-cell ‘omics in living tissues or organisms.

Metabolomics represent another powerful method to characterize and evaluate dynamic changes of the metabolic profiles present in the TME and TIME. Metabolomic approaches capture altered metabolites that can be used as biomarkers for diagnosis or assessment of treatment response, such as immune response in the context of immunotherapies. However, such an approach has some limitations to interrogating metabolism at the single cell level. To overcome some of these challenges, Wagner et al., developed an *in silico* approach to infer the cellular metabolic status based on single cell transcriptomics (268). This platform, named Compass, is an algorithm allowing network-wide deep metabolic profiling and metabolic target identification based on flux balance analysis and single cell RNA sequencing data. Applying this algorithm, the authors uncovered significant immunometabolic diversity of Th17 cells associated with multiple inflammatory effector functions. The study reported that Th17 pathogenicity is linked to a metabolic reprogramming between glycolysis and beta oxidation and that the polyamine pathway was critical for Th17 induction and restriction of Treg-like program in Th17 cells (268). In this particular example, Compass demonstrated the significance of the polyamine signaling in regulating the epigenome balancing Th17/Treg differentiation, and therefore regulating autoimmunity.

Based on the technical advancements aforementioned, the TME has been intensively analyzed in view of transcriptomic, proteomic, metabolomic, and spatial information. However, deep machine learning and AI have the potential to further our understanding by accurately integrating and managing data from these multiple -omics platforms. Newman and Alizadeh laboratories, who developed the analytical tool CIBERSORTx to impute gene expression profiles and provide estimates of abundances of mixed cell population, recently reported a new machine learning framework (EcoTyper) for the systematic identification of cell states and ecosystems integrating data from bulk RNA sequencing, single-cell RNA sequencing, and spatially-resolved expression data (269–271). The utility of EcoTyper was demonstrated by the creation of global atlases of transcriptionally distinct cellular states from sixteen types of carcinoma and revealing fundamental units of cellular organization with significance for diagnosis, disease presentation and response to therapies (270). Similarly, Steen and colleagues used this machine-learning framework to characterize the cell states and ecosystems in the TME of diffuse large B cell lymphoma (269).

## Conclusion

The role of the immune system in GBM and its interaction with tumor cells, especially GSCs, is gaining ever growing attention as both cellular compartments are critical participants of the TME, promoting disease progression and recurrence. In this review we discussed the complexity existing in GBM, with a specific focus on the diversity of CSCs and how this heterogeneity regulates the tumor immune landscape. We also analyzed the specific reciprocal relationship between these cells and how the spatial patterning of the GBM microenvironment can be regulated by the distribution and composition of CSCs and immune cells. Due to the important role of CSCs in treatment resistance and tumor recurrence, they represent an important therapeutic target. In light of their strong alliance with particular immune cells, it will be critical to develop strategies to target and disrupt communications between CSCs and immune cells, to improve recognition by cytotoxic T cells and achieve improved disease outcome.

## Author contributions

AS, DF, TG, MR, JH, MS, and LD wrote, edited and approved the submitted version of this manuscript.

## Funding

LD is supported in part by grants from the NIH (R21NS116578, 1R01NS121075), the Florida Department of Health (22L06), the Florida Center for Brain Tumor Research and Accelerate Brain Cancer Cure, the University of Florida Health Cancer Center (Pilot Grant), and the St. Baldrick’s Foundation (638733).

## Acknowledgments

**Figures 1**–**3** were created with BioRender.com.

## Conflict of interest

The authors declare that the research was conducted in the absence of any commercial or financial relationships that could be construed as a potential conflict of interest.

## Publisher’s note

All claims expressed in this article are solely those of the authors and do not necessarily represent those of their affiliated organizations, or those of the publisher, the editors and the reviewers. Any product that may be evaluated in this article, or claim that may be made by its manufacturer, is not guaranteed or endorsed by the publisher.
